# Synergistic cardioprotective effects of melatonin and deferoxamine through the improvement of ferritinophagy in doxorubicin-induced acute cardiotoxicity

**DOI:** 10.3389/fphys.2022.1050598

**Published:** 2022-11-30

**Authors:** Mira Hanna, Hanan Seddiek, Basma Emad Aboulhoda, George N. B. Morcos, Ahmed M. A. Akabawy, Marawan Abd Elbaset, Abdelsatar Abdelsatar Ibrahim, Mohamed Mansour Khalifa, Ibtesam Mahmoud Khalifah, Mostafa Said Fadel, Tarek Shoukry

**Affiliations:** ^1^ Department of Human Physiology, Faculty of Medicine (Kasr Al-Ainy), Cairo University, Egypt; ^2^ Department of Anatomy and Embryology, Faculty of Medicine, Cairo University, Cairo, Egypt; ^3^ Department of Medical Biochemistry and Molecular Biology Department, Faculty of Medicine, Cairo University, Cairo, Egypt; ^4^ Department of Basic Medical Science, Faculty of Medicine, King Salman International University, South Sinai, Egypt; ^5^ Department of Biochemistry and Molecular Biology, Faculty of Pharmacy, Helwan University, Cairo, Egypt; ^6^ Department of Pharmacology, Medical Research and Clinical Studies Institute, National Research Centre, Cairo, Egypt; ^7^ Department of Human Physiology, College of Medicine, King Saud University, Kingdom of Saudi Arabia, Riyadh, Saudi Arabia; ^8^ Department of Internal Medicine, Faculty of Medicine, Ain Shams University, Cairo, Egypt; ^9^ Department of Clinical Sciences, Faculty of Medicine, Fakeeh College for Medical Sciences, Riyadh, Saudi Arabia; ^10^ Department of Basic Medical Science, Faculty of Medicine, King Salman International University, South Sinai, Egypt

**Keywords:** ferritinophagy, ferroptosis, iron chelators, melatonin, DOX-induced cardiotoxicity

## Abstract

Ferritinophagy is one of the most recent molecular mechanisms affecting cardiac function. In addition, it is one of the pathways by which doxorubicin, one of the anticancer drugs commonly used, negatively impacts the cardiac muscle, leading to cardiac function impairment. This side effect limits the use of doxorubicin. Iron chelators play an important role in hindering ferritinophagy. Antioxidants can also impact ferritinophagy by improving oxidative stress. In this study, it was assumed that the antioxidant function of melatonin could promote the action of deferoxamine, an iron chelator, at the level of ferritinophagy. A total of 42 male Wistar rats (150–200 g) were divided into seven groups (n = 6) which consisted of group I: control normal, group II: doxorubicin (Dox), group III: melatonin (Mel), group IV: deferoxamine (Des), group V: Mel + Dox, group VI: Des + Dox, and group VII: Mel + Des + Dox. Groups III, V and VII were orally pretreated with melatonin 20 mg/kg/day for 7 days. Groups IV, VI and VII were treated with deferoxamine at a 250 mg/kg/dose once on D4 before Dox was given. Doxorubicin was given at a 20 mg/kg ip single dose. On the 8th day, the rats were lightly anaesthetized for electrocardiography analysis and echocardiography. Serum samples were collected and then sacrificed for tissue sampling. The following biochemical assessments were carried out: PCR of NCOA4, IREB2, FTH1, SLC7A11, and GPX4; and ELISA for serum cTnI, serum transferrin, tissue GSH, and malondialdehyde. In addition, histopathological assessment of heart injury; immunostaining of caspase-3, Bax, and Bcl2; and physiological function assessment by ECG and ECHO were carried out. Doxorubicin-induced acute significant cardiac injury with increased ferritinophagy and apoptosis responded to single and combined prophylactic treatment, in which the combined treatment showed mostly the best results. In conclusion, using melatonin as an antioxidant with an iron chelator, deferoxamine, could hinder the hazardous cardiotoxic effect of doxorubicin. However, further studies are needed to detect the impact of higher doses of melatonin and deferoxamine with a prolonged treatment period.

## 1 Introduction

Doxorubicin (Dox) is a very effective chemotherapeutic drug in various cancer types; however, its use is limited due to its cardiotoxicity, which may start with arrhythmia and end up with congestive heart failure (Bilginoğlu et al., 2014). Dox-induced cardiotoxicity depends mainly on the induction of oxidative stress and increases reactive oxygen species (ROS) production and lipid peroxidation and decreases endogenous antioxidants. This induces the intrinsic mitochondrion-dependent apoptotic pathway in cardiomyocytes (Öz et al., 2006; Zhang et al., 2009; Octavia et al., 2012). Fang et al. (2019) showed that the inhibition of ferroptosis protects the heart against doxorubicin-induced cardiomyopathy (Fang et al., 2019).

Ferroptosis, iron-dependent cell death, is characterized by the loss of cellular redox homeostasis and unchecked lipid peroxidation. It plays a role in the induction and/or progression of several pathological conditions of different organs, including the heart (Gao et al., 2015; Angeli et al., 2017). At the same time, iron is important for cardiac metabolic requirements. Cardiac muscle depends on iron as a permanent source of energy and cofactor for many of its enzymes. Yet, persistent blood perfusion to cardiac muscle predisposes it to iron overload, oxidative stress, and lipid peroxidation. Together, it can trigger ferroptosis mechanisms in cardiac muscle due to the disturbance of intracellular iron balance and redox activities. The maintenance of balanced iron homeostasis depends on many iron transport and storage mechanisms, as well as redox balance that relies mainly on glutathione and glutathione peroxidase (GPX4) (Hirschhorn and Stockwell 2019). However, the pathophysiological effect of ferroptosis on cardiomyocyte death is still not fully studied. In addition, to date, there are no therapeutic methods that regain cardiac function after cardiomyocyte death in pathological settings. Consequently, the targeted policy is to prevent cardiomyocyte death due to severe cardiac injury, as in cardiomyopathy. Furthermore, the inhibition of ferroptosis is considered an innovative way to prevent cardiac cell death.

Another important issue is that the drugs designed to regain cardiac functions after cardiomyocyte death have fallen short of high expectations. Thus, the prevention of cardiomyocyte death before the actual occurrence of cardiomyopathy could present a more reliable treatment modality. Modulating ferroptosis in the context of cardiomyocyte injury provides a fairly coherent set of ideas about the feasibility of introducing iron-chelating drugs as potentially promising protective and/or therapeutic strategies in cardiac diseases.

Accordingly, iron chelators may play an effective role in protecting the cardiac muscle cells from the effect of ferroptosis and iron overload. Deferoxamine (Des) is a well-known iron chelator used to treat iron overload, especially in hematological disorders (Pennell et al. 2006). Studies have shown that Des decreases the production of intracellular ROS. Hence, it inhibits ferroptosis cell death (Kwon et al. 2015; Ooko et al. 2015). Moreover, Des protected the cardiac muscle cells against the perturbation of cardiac ECG in a gerbil model of iron overload cardiomyopathy (Obejero-Paz et al. 2003). The prolonged record of Des therapeutic use in treating iron overload accompanying hematological disorders provides possible treatment in preventing ferroptosis during acute myocardial injury.

Furthermore, melatonin (Mel) is an important natural antioxidant secreted by the pineal gland. It plays different biological roles related to circadian rhythms and cardiovascular, neuroendocrine, and immune functions based on its antioxidant properties (Öz et al., 2006). Its ability to scavenge free radicals depends on directly detoxifying them through electron donation. Consequently, it may reduce Dox-induced oxidative stress, protecting against its toxicity by inhibiting ROS production (Zhang et al., 2013), restoring the activity of the antioxidative enzymes in the cardiac muscle, and modulating iron, ferritin, and transferrin levels (Othman et al., 2008).

The present study aimed to detect the protective effect of adjuvant therapy of melatonin with deferoxamine as a cardioprotective agent in the case of Dox-induced acute cardiotoxicity in rats by preserving iron homeostasis in cardiac tissue.

## 2 Materials and methods

### 2.1 Animals

A total of 42 male Wistar rats weighing 150–200 g were purchased from the National Research Centre animal house. The animal ethics committee of Cairo University approved all experimental procedures (approval no: CU III F 14 20). All animals were housed in a temperature-controlled (23 ± 12°C) environment under a 12-h light/dark cycle and 50% humidity and with free access to tap water and a standard laboratory pellet diet.

### 2.2 Experimental design

The animals were randomly divided into seven groups of six rats per group (n = 6 × 7):

Group I: Control group in which saline 0.9% was administered intraperitoneally (ip) instead of Dox and Des, while oral gavage of tween 80 and distilled water instead of melatonin.

Group II: Dox group in which a single dose of Dox was administered ip.

Group III: Mel group (a cohort group to show the effect of melatonin alone).

Group IV: Des (a cohort group to show the effect of deferoxamine alone).

Group V: Mel + Dox (a single pretreated group with melatonin for 7 days before Dox injection on day 4).

Group VI: Des + Dox (a single pretreated group with deferoxamine 30 min before Dox injection on day 4).

Group VII: Mel + Des + Dox (a combined pretreated group with melatonin for 7 days and deferoxamine 30 min before Dox injection on day 4).

### 2.3 Drugs used

Doxorubicin (adricin manufactured by Hikma Specialized Pharmaceuticals) 50 mg/25 ml was administered in 20 mg/kg body weight through a single intraperitoneal (ip) injection.

Deferoxamine 0.5 g vial (desferal manufactured by Novartis) was dissolved in saline and injected at 250 mg/kg, ip.

Melatonin 10 mg capsules (Puritan’s Pride) 20 mg/kg were given daily by oral gavage (Mortazavi et al., 2017; Aygun and Gul, 2019). Melatonin was prepared by adding Tween 80 at a concentration of 10 mg/ml (Elshall et al., 2022) and then distilled water (Shokrzadeh et al., 2014; Mahmoud and El-Deek, 2019) at a concentration of 2.4 mg/ml (Supplementary Figure S9).

Melatonin was administered for 7 days; deferoxamine was given on day 4, 1 h after Mel and 30 min before Dox were given.

**FIGURE 1:**
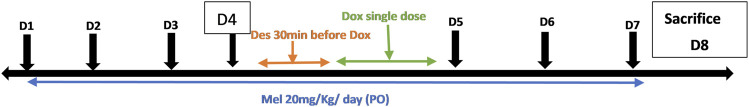
Timeline of drug administration throughout the experiment. Dox, doxorubicin; Mel, melatonin; Des, deferoxamine.

The timeline of drug administration is precisely shown in Figure 1. By the end of the experiment, the rats were sacrificed by head dislocation, and the serum and heart tissue samples were collected and kept at −80°C until analyses were performed.

### 2.4 Electrocardiography

At the end of the treatment protocol, animals were anaesthetized and sedated with a combination of ketamine (100 mg/kg) and xylazine (10 mg/kg) ip. During ECG recordings, the rats were placed on a warming blanket to avoid hypothermia. In all animals, 10 min after anesthesia, three needle electrodes were inserted under the skin of the animals for limb lead II. ECG parameters were recorded for 3 min using the PowerLab system (AD Instruments) connected to a PC running LabChart 7 for each rat separately. The changes in the R-R interval (sec), heart rate (bpm), QT interval (sec), R amplitude (mV), and ST-segment amplitude (mV) were determined.

### 2.5 Echocardiography

Echocardiography (Esaote MyLab) was performed using the MyLab 30 Vet Gold Phased array probe 10 MHz in lightly anesthetized rats with ketamine (50 mg/kg) and xylazine (1 mg/kg) ip. Systolic function was analyzed by ejection fraction (EF), fractional shortening (FS), left ventricular end-diastolic diameter (LVEDd), and left ventricular end-systolic diameter (LVESd). All parameters were obtained from a short-axis view.

### 2.6 Biochemical assays and measurements of

#### 2.6.1 Serum cardiac troponin I (cTnI) and serum transferrin

At the end of the experiment, all rats were deeply anesthetized under anesthesia, and their blood samples were collected in tubes. After 30 min, these tubes were centrifuged at 3,000 rpm for 10 min; sera were separated and stored at −80°C for the biochemical measurement of cardiac troponin I (cTnI) and serum transferrin levels using the sandwich ELISA technique, according to the manufacturer’s protocols. Measurement was carried out at max using an ELISA plate reader (Stat Fax 2200, Awareness Technologies, Florida, United States).

#### 2.6.2 Tissue malondialdehyde and glutathione

After the animals’ sacrifice, tissues were dissected, washed thoroughly, and rinsed with ice. They were gently blotted between the folds of a filter paper and weighed in an analytical balance. Next, 10% homogenate was prepared in 0.05 M phosphate-buffered saline (pH 7) using a polytron homogenizer at 4°C.

The homogenate was centrifuged at 10,000 rpm for 20 min to remove the cell debris. Then, the supernatant (tissue homogenate) was separated and aliquoted. According to manual instructions, a fresh homogenate aliquot was used to determine MDA and GSH levels using a colorimetric assay kit. In contrast, the aliquot was stored at −80°C for PCR analyses.

#### 2.6.3 Quantitative real-time PCR analysis

From freshly homogenized cardiac tissues, the total RNA was extracted using the Direct-zol RNA Miniprep Plus Kit (Cat# R2072, Zymo Research Corp., United States). The quantity and quality were assessed using a Beckman dual spectrophotometer (United States).

A SuperScript IV One-Step RT-PCR kit (Cat# 12594100, Thermo Fisher Scientific, Waltham, MA, United States) was utilized for reverse transcription of extracted RNA, followed by PCR. A 96-well plate StepOne instrument (Applied Biosystems, United States) was used in a thermal profile as follows: 10 min at 45°C for reverse transcription, 2 min at 98°C for RT inactivation and initial denaturation by 40 cycles of 10 s at 98°C, and 10 s at 55°C and 30 s at 72°C for the amplification step. After the RT-PCR run, the data were expressed in the cycle threshold (Ct) for the target genes and housekeeping genes. Duplicate analysis was adopted. Normalization for variation in the expression of target genes (Cat. No.), Ncoa4 (QT01799378), Ireb2 (QT00177716), Slc7a11 (QT00393841), Gpx4 (QT01169434), and *F*th1 (QT01817844), was performed referring to the mean critical threshold (CT) expression values of the GAPDH (QT00199633) housekeeping gene by the ΔΔCt method. The relative quantitation (RQ) of each target gene is quantified according to the calculation of the 2^−ΔΔCT^ method. All primers used throughout the experiment were ready-to-use QuantiTect ^®^ Primer Assay kits purchased from Qiagen.

### 2.7 Histopathological analysis

Histological examination of the myocardium was performed through routine histological procedures *via* fixation, dehydration, clearing, and paraffin embedding. In addition, 5-μm sections were cut and stained with hematoxylin and eosin for microscopic evaluation.

Determination of cardiac myocyte degeneration in the left ventricular heart section was scored on a scale of 0–3, as previously described (Singla et al., 2012; Guo et al., 2018), where no signs of myofibrillar degeneration were graded as 0. Score 1 is given when <5% of cells exhibit early myofibrillar loss. Grade 2 is scored when 15%–30% of cells exhibit marked myofibrillar loss and/or cytoplasmic degeneration. Diffuse >30% cell damage, with most cardiac myocytes indicating myofibrillar disruption and marked loss of contractile elements, was graded as 3.

### 2.8 Immunohistochemical analysis

Immunohistochemical staining with rabbit polyclonal anti-Bax (ab53154), anti-Bcl2 (ab59348), and anti-caspase-3 (ab44976) primary antibodies (IHC-P, at 1:100 dilution, Abcam^®^, Cambridge, MA, United States) was performed *via* the Dako automated system (EnVision FLEX Peroxidase-blocked), as previously described (El Asar et al., 2019). In addition, the goat anti-rabbit IgG H&L (HRP) was added as the secondary antibody. Negative controls were obtained by omitting the primary antibodies in the automated staining protocol. The area percentage of the positive immune reaction of Bax, Bcl2, and caspase-3 was estimated using Leica Qwin 500C, Leica Image Analysis System Ltd. (Cambridge, England), in 10 live random fields (×400 magnification) from each group. The sections were evaluated using a Leica ICS150 HD microscope camera (Leica Imaging, Cambridge, United Kingdom).

### 2.9 Statistical analysis

Statistical analysis was performed by SPSS software (version 17.0; SPSS, United States). The parametric variable groups were compared using one-way ANOVA, followed by the Tukey *post hoc* test. The Kruskal–Wallis test compared the groups of nonparametric variables. All the values are presented as the mean ± standard error of the mean (SEM). Graphs were sketched by GraphPad Prism (United States) version 7 software. The values of *p* < 0.05 were considered to indicate a statistically significant difference.

## 3 Results

### 3.1 Results of the biochemical assay of

#### 3.1.1 Cardiac troponin I (cTnI)

**FIGURE 2:**
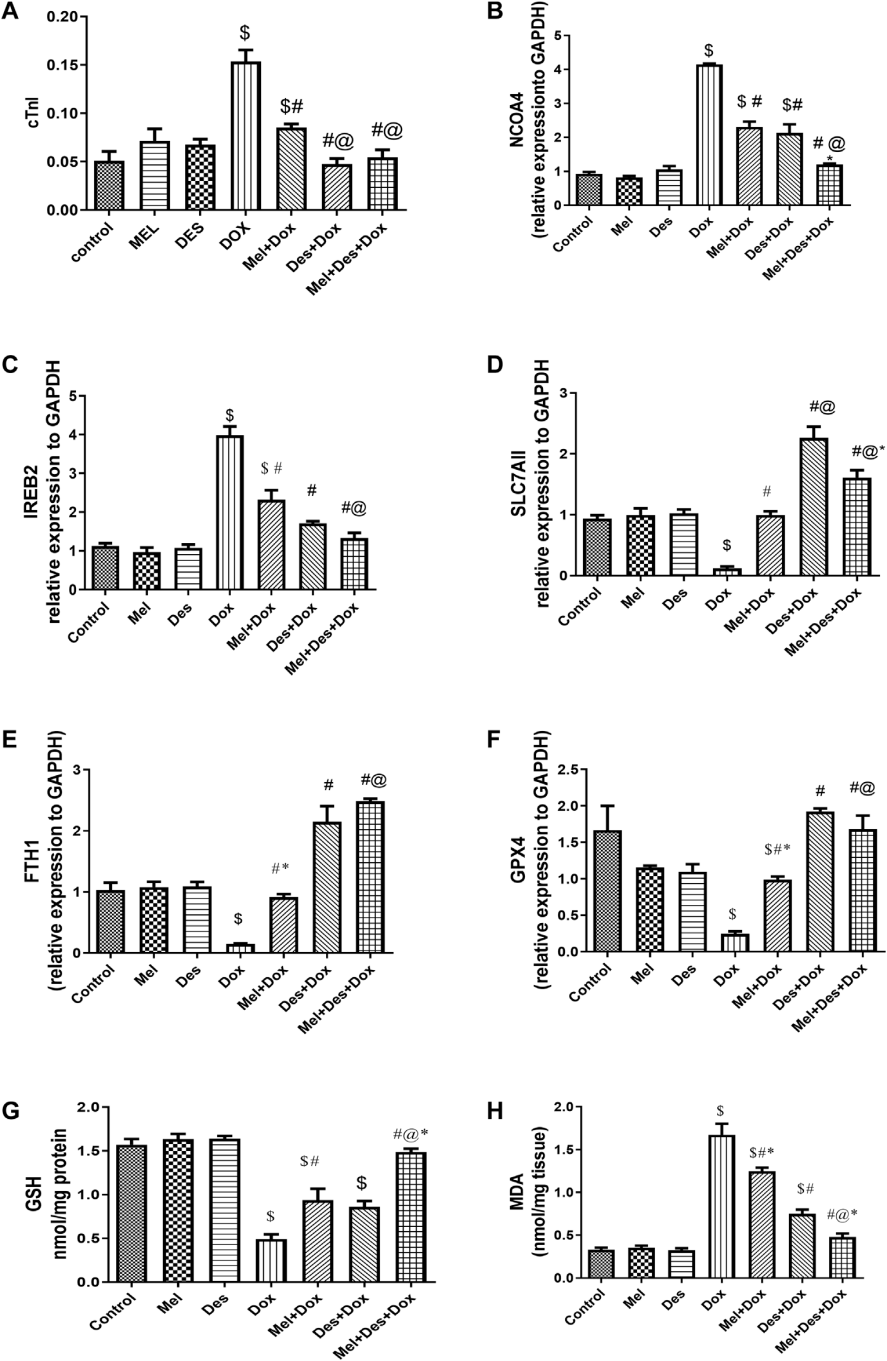
**(A–H)** Biochemical assay results for cTnI, NCOA4, IREB2, SLC7AII, FTH1, GPX4, GSH, and MDA, respectively. $, significant *versus* control; #, significant *versus* Dox; @, significant *versus* Mel-Dox; *, significant *versus* Des-Dox. Mel, melatonin; Des, deferoxamine; Dox, doxorubicin.

Biochemical analysis of cTnI serum levels showed significant improvement in cTnI serum levels in the treated groups Des-Dox, Mel-Dox, and Mel-Des-Dox than the diseased Dox group Dox, in which the Des-Dox group showed the best improvement. However, there was no significant difference between groups Des-Dox and Mel-Des-Dox compared with the control group. However, there was a substantial difference between Mel-Dox and control groups. This shows that for groups treated with Des alone or Mel, Des led to returning cTnI to the control level, while Mel only did not. In addition, there was no significant difference between the Des-Dox and the Mel-Des-Dox groups (Figure 2A and Supplementary Table S1).

#### 3.1.2 Downregulation of NCOA4

Quantitative PCR analysis of mRNA NCOA4 tissue levels showed significant downregulation in the treated groups Des-Dox, Mel-Dox, and Mel-Des-Dox compared to the diseased Dox group, in which the Mel-Des-Dox group showed the best improvement. There was no significant difference between Mel-Des-Dox and the control group. However, there was a significant difference between groups Des-Dox and Mel-Dox compared with the control. There was no significant difference between Mel-Dox and Des-Dox groups, but there was considerable improvement in the Mel-Des-Dox group compared with Mel-Dox and Des-Dox groups. This showed that groups treated with Mel and Des returned the NCOA4 tissue level to the control level, while Mel and Des only did not, despite their improvement level (Figure 2B and Supplementary Table S1).

#### 3.1.3 Downregulation of IREB2

Quantitative PCR analysis of mRNA IREB2 tissue levels showed a significant improvement in a downregulation form in the treated groups Des-Dox, Mel-Dox, and Mel-Des-Dox compared with the diseased Dox group, in which the Mel-Des-Dox group showed the best improvement. There was no significant difference between Des-Dox and Mel-Des-Dox groups compared with the control group. However, there was a substantial difference between the Mel-Dox group and the control. There was no significant difference between Mel-Dox and Des-Dox, but there was considerable improvement between Mel-Des-Dox and Mel-Dox groups. This showed that for groups treated with both Mel and Des, the group treated with Des only led to returning the IREB2 tissue level to the control level. At the same time, Mel only did not despite its improvement level (Figure 2C and Supplementary Table S1).

#### 3.1.4 Upregulation of SLC7A11

Quantitative PCR analysis of mRNA SLC7A11 tissue levels showed significant upregulation improvement in the treated groups Des-Dox, Mel-Dox, and Mel-Des-Dox compared with the diseased Dox group, in which group Des-Dox showed the best improvement. There was a significant difference between Des-Dox and Mel-Des-Dox groups compared with the control group, in which Des-Dox and Mel-Des-Dox groups showed better results than the control. However, there was no significant difference between the Mel-Dox group and the control. There was a substantial difference between Mel-Dox and Des-Dox and Me-Des-Dox, in which they are better, and there was significant improvement between Des-Dox and Mel-Des-Dox. This showed that all treated groups returned the SLC7A11 tissue level to the control level or better, while the Des-Dox group showed the best result (Figure 2D and Supplementary Table S1).

#### 3.1.5 Upregulation of FTH1

Quantitative PCR analysis of mRNA FTH1 tissue levels showed a significant improvement (increase/upregulation) in the treated groups Des-Dox, Mel-Dox, and Mel-Des-Dox compared with the diseased Dox group, in which the Mel- Des-Dox group showed the best improvement. There was a significant difference between Des-Dox and Mel-Des-Dox groups compared with the control group, in which Des-Dox and Mel-Des-Dox groups showed better results than the control. However, there was no significant difference between the Mel-Dox group and the control. There was a considerable difference between Mel-Dox and Des-Dox and Mel-Des-Dox, in which they are better, and there was no significant difference between Des-Dox and Mel-Des-Dox. This showed that all treated groups replenished the SLC7A11 tissue level to the control level or better, while the Mel-Des-Dox group showed the best result (Figure 2E and Supplementary Table S1).

#### 3.1.6 Upregulation of GPX4

Quantitative PCR analysis of mRNA GPX4 tissue levels showed significant upregulation improvement in the treated groups Des-Dox, Mel-Dox, and Mel-Des-Dox compared with the diseased Dox group, in which the Des-Dox group showed the best improvement. There was a significant difference between the Mel-Dox and the control groups. However, there was no significant difference between Des-Dox and Mel-Des-Dox groups compared with the control. On the other hand, there was a substantial difference between Des-Dox and Mel-Dox, in which Des-Dox is better. This showed that for all treated groups, there was a significant improvement in which the Des-Dox group and Mel-Des-Dox showed the best results returning to the control level (Figure 2F and Supplementary Table S1).

#### 3.1.7 GSH

Biochemical analysis of GSH tissue levels showed significant improvement in the treated groups Mel-Dox and Mel-Des-Dox compared with the diseased Dox group, in which the Mel-Des-Dox group showed the best improvement. In contrast, Des-Dox showed nonsignificant improvement. Furthermore, there was a significant difference between Des-Dox and Mel-Dox groups compared with the control group, while Mel-Des-Dox showed no significant difference compared with the control. In addition, there was a significant difference between the Mel-Des-Dox group and Mel-Dox and Des-Dox groups. This showed that the treated group with Mel-Des-Dox returned the GSH tissue level to the control level (Figure 2G and Supplementary Table S1).

#### 3.1.8 MDA

Biochemical analysis of MDA tissue levels showed significant improvement in the treated groups Des-Dox, Mel-Dox, and Mel-Des-Dox compared with the diseased Dox group in which the Mel-Des-Dox group showed the best improvement. There was a significant difference between Des-Dox and Mel-Dox groups compared with the control group, while Mel-Des-Dox showed no significant difference compared with the control. In addition, there was a considerable difference between the Mel-Des-Dox group and Mel-Dox and Des-Dox groups. This showed that the group treated with Mel-Des-Dox returned the MDA tissue level to the control level (Figure 2H and Supplementary Table S1).

#### 3.1.9 Serum transferrin

Biochemical analysis of transferrin serum levels showed no significant differences between the groups (Supplementary Table S1).

### 3.2 Result of the histopathological assay

#### 3.2.1 Histopathological results

Examination of the hematoxylin and eosin-stained sections showed numerous degenerative myocardial changes in the form of cardiomyocyte fragmentation, blood extravasation, and dense interstitial inflammatory cellular infiltration. In addition, hemovascular changes were also revealed as vascular dilatation, capillary congestion, and interstitial edema in between the disrupted cardiomyocytes.

**FIGURE 3:**
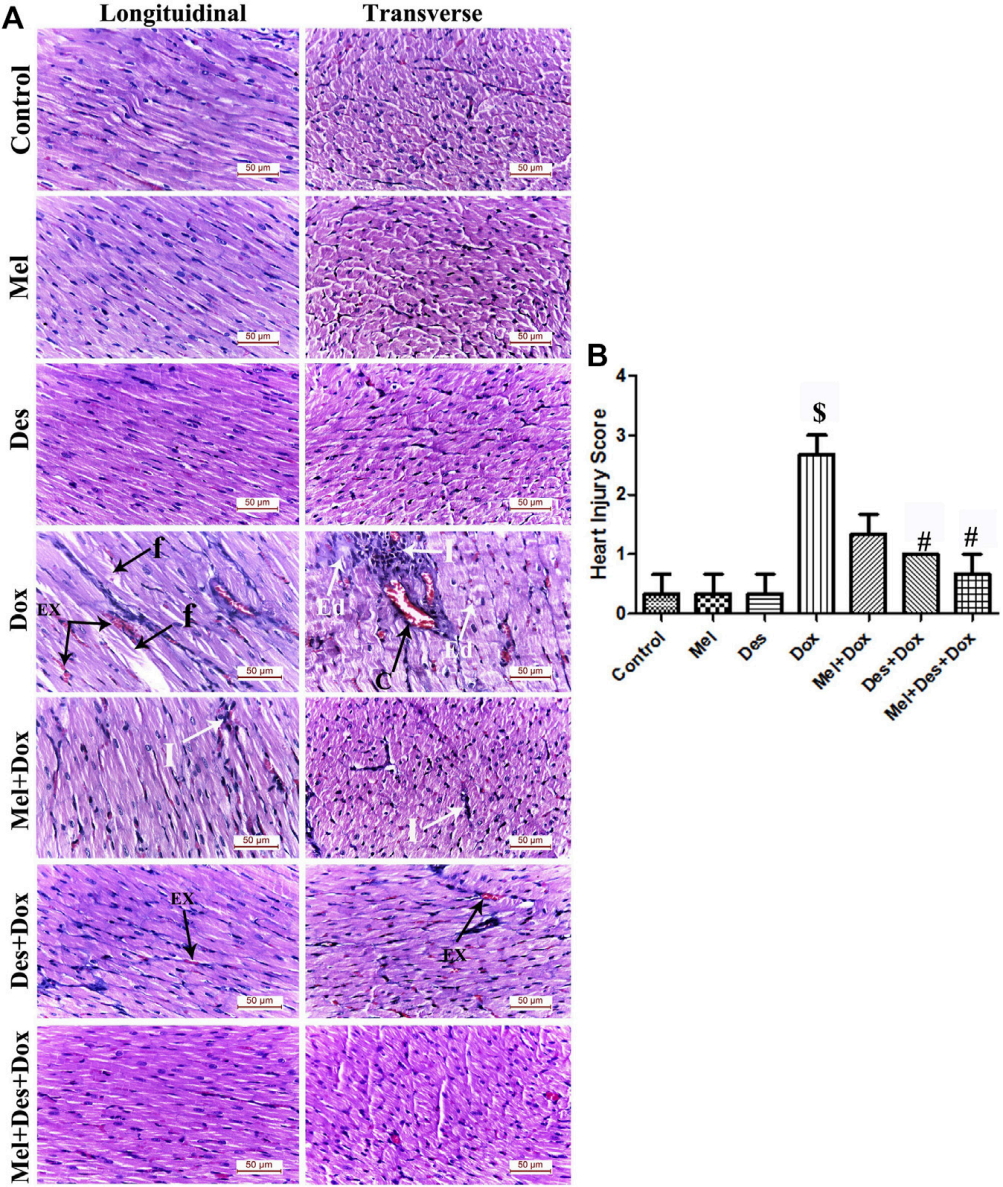
**(A)** Representative figures of H&E-stained sections of the different study groups displaying normal cardiac myocytes in the control, Mel, and Des groups. The Dox group shows focal degeneration of the cardiac muscle fibers with cardiomyocyte fragmentation (f) and blood extravasation (Ex). Granulation tissue infiltrated with dense inflammatory infiltration (I) formed of lymphocytes, neutrophils, and macrophages is also observed. Dilated congested capillaries (C) and interstitial edema (Ed) in-between the disrupted cardiomyocytes are also observed. The Mel-Dox group shows minimal myocyte disruption with residual mononuclear inflammatory cellular infiltration (I). The Des-Dox group shows regularly arranged cardiac muscle fibers with minimal blood extravasation (Ex). The combined Mel-Des-Dox group shows improved architecture of the cardiac muscle fibers where they exhibit normal acidophilic sarcoplasm, central oval vesicular cardiomyocyte nuclei, and peripheral flat spindle-shaped nuclei of fibroblasts. The cardiomyocytes appear separated by narrow inter-fibrillar spaces and healthy blood capillaries. (Scale bar 50 µm). **(B)** Heart injury score of the different study groups. ($: significant *versus* control, #: significant *versus* Dox, @: significant *versus* Mel-Dox, *: significant *versus* Des-Dox at *p* < 0.05, (n = 10) using ANOVA and Tukey’s *post hoc* test for pairwise comparison.). Mel: melatonin; Des: deferoxamine; Dox: doxorubicin.

The Mel-Dox group showed minimal myocyte disruption with residual mononuclear inflammatory cellular infiltration. The Des-Dox group showed improvement in the myocardial structure, where the cardiac muscle fibers appeared regularly arranged with only minimal blood extravasation. The combined (Mel-Des-Dox) group showed markedly improved architecture of the cardiac muscle fibers, where they exhibited normal acidophilic sarcoplasm and central vesicular nuclei. The cardiomyocytes were observed to be separated by narrow inter-fibrillar spaces and healthy blood capillaries. Evaluation of the heart injury score revealed significant improvement in the treated groups Des-Dox, Mel-Dox, and Mel-Des-Dox compared with the diseased Dox group, in which the Mel-Des-Dox group showed the best improvement. There was no significant difference between all the treated groups and the control group and compared with each other (Figure 3 and Supplementary Table S2).

#### 3.2.2 Immunohistochemical analysis of caspase-3

**FIGURE 4:**
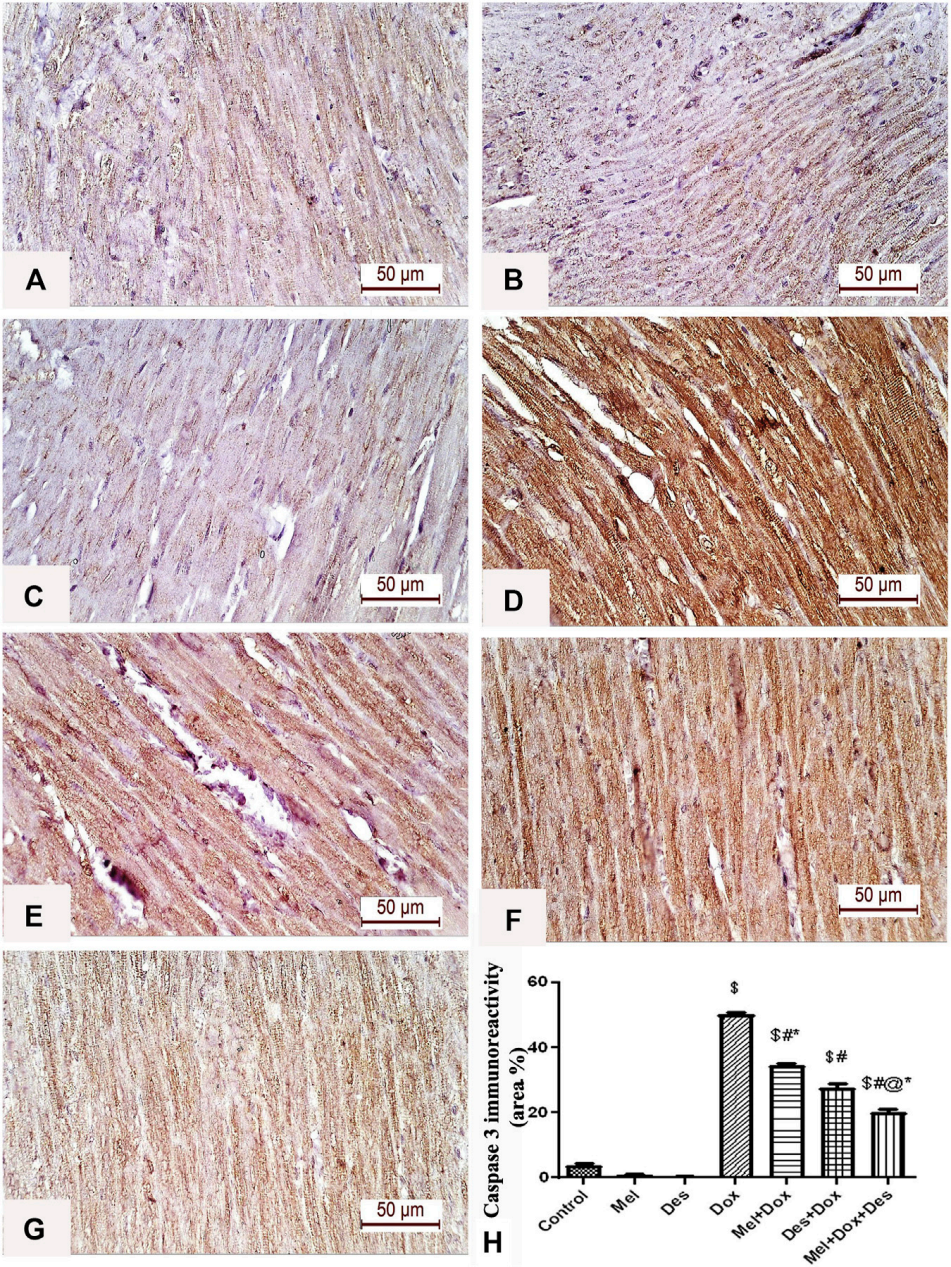
Representative figures of caspase-3 immunohistochemical expression in the myocardium. **(A)** Control group, **(B)** Mel, **(C)** Des show negative caspase-3 immunoreactivity in the myocardial fibers. **(D)** Dox group shows a strong positive caspase-3 immune reaction in the cardiac myofibrils. **(E)** Mel-Dox and **(F)** Des-Dox show a moderate caspase-3 immune reaction in the cardiomyocytes. **(G)** Minimal immunoreactivity is observed in the combined Mel and Des-treated group (Mel-Dox-Des) (scale bar 50 µm). **(H)** Area percentage of caspase-3 immunohistochemical expression in the different study groups, $: significant *versus* control, #: significant *versus* Dox, @: significant *versus* Mel-Dox, *: significant *versus* Dox-Des at *p* < 0.05, (n = 10) using ANOVA and Tukey’s *post hoc* test for pairwise comparison. Mel: melatonin; Des: deferoxamine; Dox: doxorubicin.

Quantitative immune-histopathological analysis of caspase-3 showed significant improvement in the treated groups Des-Dox, Mel-Dox, and Mel-Des-Dox than the diseased Dox group, in which the Mel-Des-Dox group showed the best improvement. However, there was still a significant difference between Des-Dox, Mel-Dox, and Mel-Des-Dox groups compared with the control. Furthermore, there was a significant difference between Mel-Dox and Des-Dox, in which Des-Dox showed better improvement. In addition, there was significant improvement (decrease) in Mel-Des-Dox compared with Mel-Dox and Des-Dox. This showed that for treated groups, whether with both Mel and Des or each one alone, led to the improvement of caspase-3 expression. However, the Mel-Des-Dox group showed the best improvement but did not return to the control level (Figure 4 and Supplementary Table S2).

#### 3.2.3 Immunohistochemical analysis of Bax and Bcl2

Quantitative immunohistochemical analysis of Bax showed significant improvement (decrease) in the treated groups Des-Dox, Mel-Dox, and Mel-Des-Dox compared to the diseased Dox group in which group Mel-Des-Dox showed the best improvement. However, there was still a significant difference between Des-Dox, Mel-Dox, and Mel-Des-Dox groups compared with the control. Furthermore, there was a significant difference between Mel-Dox and Des-Dox, in which Des-Dox showed better improvement. In addition, there was significant improvement (decrease) in Mel-Des-Dox compared with Mel-Dox and Des-Dox. This showed that treated groups, whether with both Mel and Des or each one alone, led to the improvement of Bax expression, and the Mel-Des-Dox group showed the best improvement while not returning to the control level.

**FIGURE 5:**
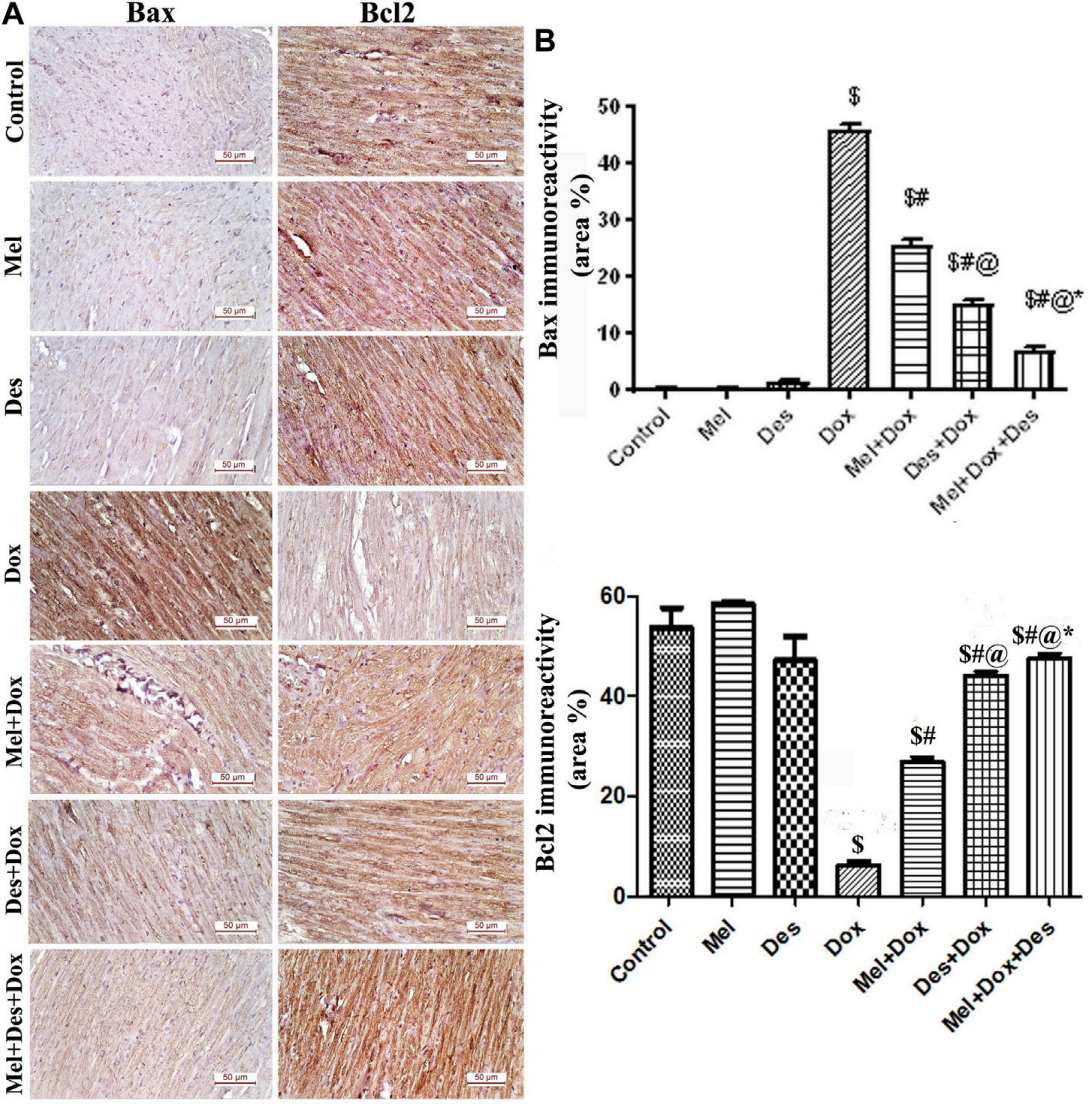
**(A)** Representative figures of Bax/Bcl2 immunoreactivity in the myocardium (scale bar 50 µm). **(B)** Area percentage of Bax/Bcl2 immunohistochemical expression in the different study groups, $: significant *versus* control, #: significant *versus* Dox, @: significant versus Mel-Dox, *: significant versus Des-Dox at *p* < 0.05, (n = 10) using ANOVA, Tukey post-hoc test for pairwise comparison. Mel: melatonin, Des: Deferoxamine, Dox: Doxorubicin.

Quantitative immunohistochemical analysis of Bcl2 showed significant improvement in the treated groups Des-Dox, Mel-Dox, and Mel-Des-Dox compared with the diseased Dox group [6.278 (±2.652)] in which the Mel-Des-Dox group showed the best improvement. There was a significant difference between Des-Dox and Mel-Dox groups compared with the control group, in which treated groups showed better results than the control, and Mel-Des-Dox was the best. There was a significant difference between Des-Dox and Mel-Dox compared with Mel-Des-Dox. This showed that treated groups, whether with both Mel and Des or each one alone, led to the improvement of Bcl2 expression, and the Mel-Des-Dox group showed the best improvement (Figure 5 and Supplementary Table S2).

### 3.3 Results of the physiological function assessment

#### 3.3.1 Electrocardiography (ECG)

**FIGURE 6:**
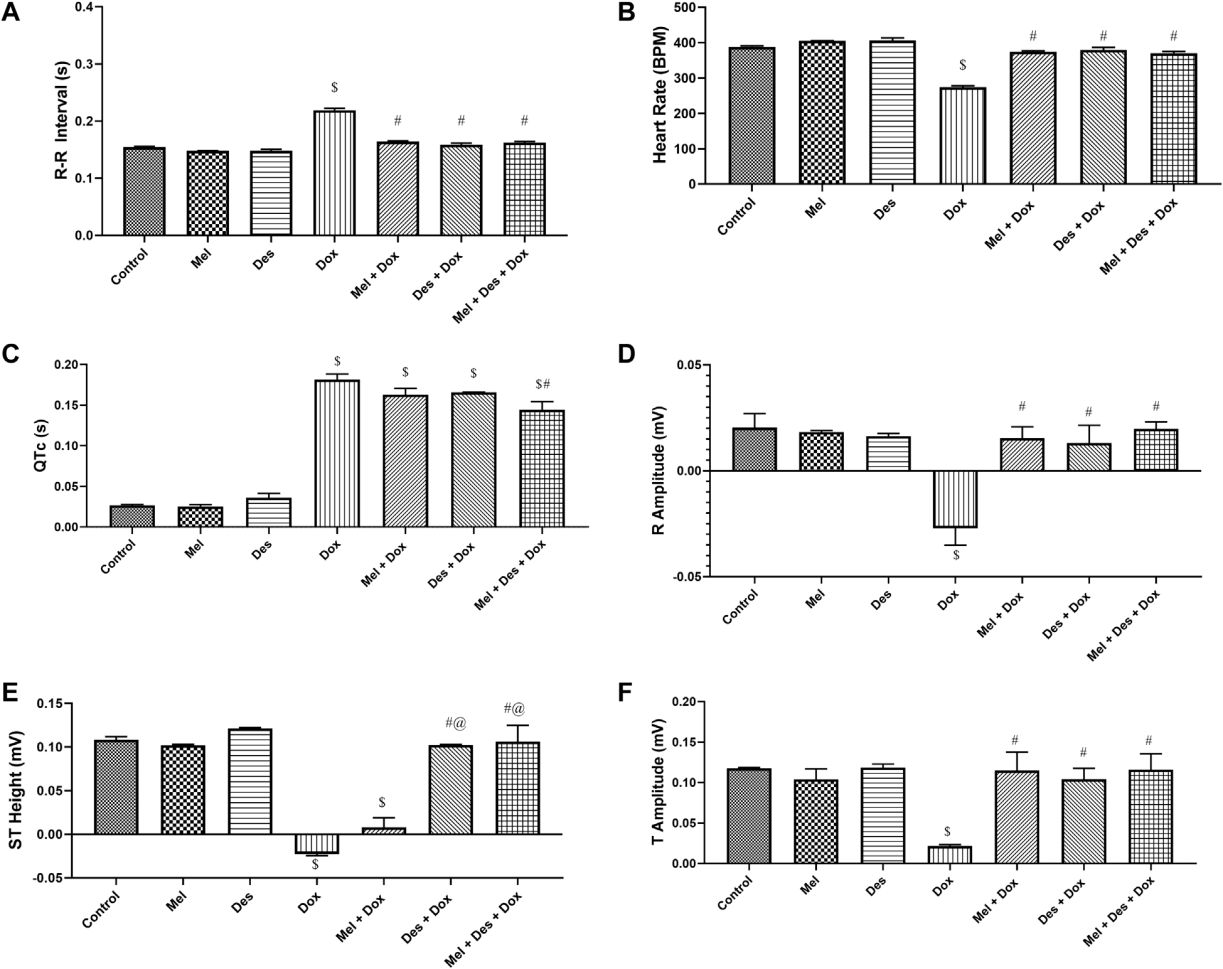
Graphs showing the statistical difference between groups regarding ECG measurements. $: significant *versus* control, #: significant *versus* Dox, @: significant *versus* Mel-Dox, *: significant *versus* Des-Dox. Mel: melatonin; Des: deferoxamine; Dox: doxorubicin.

The control and experimental groups ' ECG patterns (RR interval duration and, hence, heart rate, R amplitude, QTc interval, ST-segment amplitude, and T amplitude) are shown in Figure 6 (and Supplementary Figure S8).

##### 3.3.1.1 R-R interval (s)

The ECG pattern regarding the R-R interval showed significant improvement in the treated groups Des-Dox, Mel-Dox, and Mel-Des-Dox compared with the diseased Dox group, which showed decreased R-R interval. However, there was no significant difference between all the treated and the control groups. This showed that all treated groups displayed improvement in the R-R interval back to the control level (Supplementary Table S3).

##### 3.3.1.2 Heart rate (bpm)

Consequently, regarding the heart rate, it showed the same results depending on the R-R interval, in which all treated groups, Des-Dox, Mel-Dox, and Mel-Des-Dox, displayed significant improvement compared with the diseased Dox group which showed a decreased heart rate. There was no significant difference between all treated and the control groups. This showed that all treated groups improved the heart rate to the control level (Supplementary Table S3).

##### 3.3.1.3 QTc interval (s)

The QTc interval showed improvement in the treated groups Des-Dox, Mel-Dox, and Mel-Des-Dox compared with the diseased Dox group which showed a prolonged QTc interval. Mel-Des-Dox was the only group that significantly improved compared with the Dox group. However, all treated groups were appreciably prolonged compared with the control level, with Mel-Des-Dox showing the best result (Supplementary Table S3).

##### 3.3.1.4 R amplitude (mV)

The R amplitude showed significant improvement in the treated groups Des-Dox, Mel-Dox, and Mel-Des-Dox compared with the diseased Dox group that showed decreased R amplitude. However, there was no significant difference between all treated and the control groups. This showed that all treated groups improved the R amplitude back to the control level (Supplementary Table S3).

##### 3.3.1.5 ST segment height (mV)

The ST segment amplitude showed improvement in the treated groups Des-Dox, Mel-Dox, and Mel-Des-Dox compared with the diseased Dox group that showed a depressed ST segment. Mel-Des-Dox and Des-Dox groups showed significant improvement compared with the Dox group, while the Mel-Dox group did not. There was no significant difference between Mel-Des-Dox and Des-Dox groups compared with the control level, with Mel-Des-Dox showing the best result (Supplementary Table S3).

##### 3.3.1.6 T amplitude (mV)

T amplitude showed significant improvement in the treated groups Des-Dox, Mel-Dox, and Mel-Des-Dox, with the diseased Dox group showing decreased T amplitude. However, there was no significant difference between all treated and the control groups. This led to all treated groups displaying improvement in the T amplitude back to the control level (Supplementary Table S3).

#### 3.3.2 Echocardiography

**FIGURE 7:**
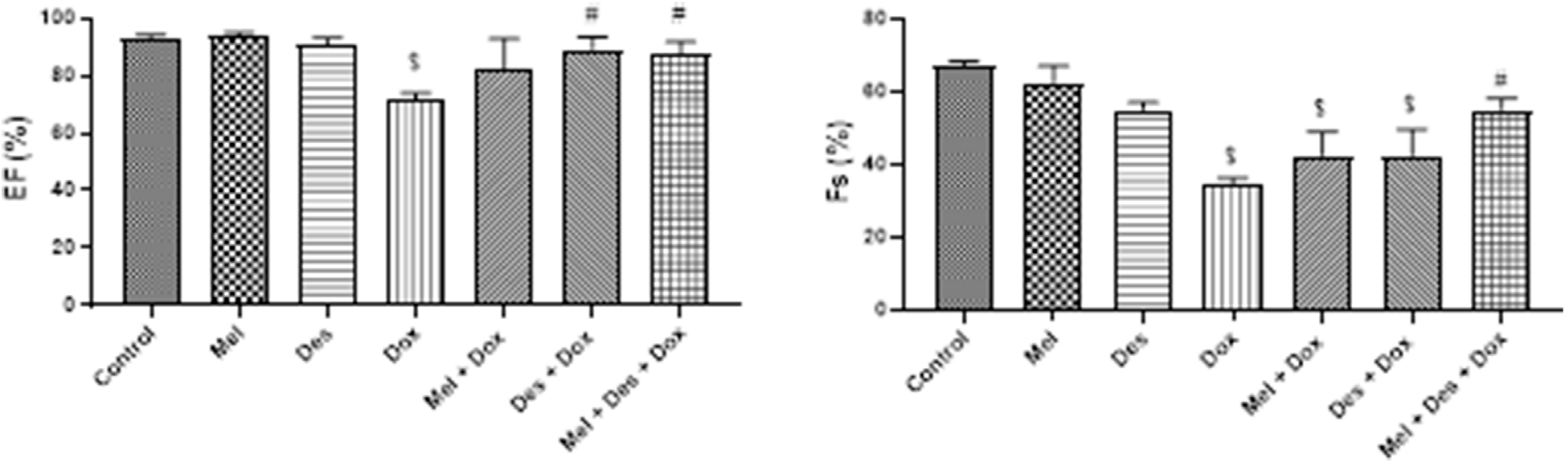
Graphs showing the results of ejection fraction and fractional shortening. $: significant *versus* control, #: significant *versus* Dox, @: significant *versus* Mel-Dox, *: significant *versus* Des-Dox. Mel: melatonin; Des: deferoxamine; Dox: doxorubicin.

The left ventricular function was assessed by measuring the ejection fraction (EF) and fractional shortening (FS) (Figure 7)

##### 3.3.2.1 Regarding ejection fraction

EF showed improvement in the treated groups Des-Dox, Mel-Dox, and Mel-Des-Dox compared to the diseased Dox group, which showed decreased EF. Mel-Des-Dox and Des-Dox showed significant improvement compared with the Dox group, while the Mel-Dox group did not. There was no significant difference between the treated groups compared with the control level (Supplementary Table S4).

##### 3.3.2.2 Regarding fractional shortening

FS showed improvement in the treated groups Des-Dox, Mel-Dox, and Mel-Des-Dox compared with the diseased Dox group that showed decreased FS. Mel-Des-Dox showed significant improvement compared with the Dox group, while Mel-Dox and Des-Dox groups did not. There was no significant difference between Mel-Des-Dox and the control level (Supplementary Table S4).

## 4 Discussion

The present study showed the following important findings: 1) doxorubicin (Dox) exerts its acute cardiotoxic effect through the ferritinophagy–ferroptosis pathway; 2) Dox increases oxidative stress and lipid peroxidation, upregulating ferritinophagy-inducing genes (NCOA4 and IREB2), and downregulating ferritinophagy inhibitors (SLC7A11 and FTH1); 3) starting pretreatment with melatonin as an antioxidant before Dox injection with continuation for another 4 days could hinder Dox-induced acute cardiotoxicity; 3) pretreatment with deferoxamine, as an iron chelator, 30 min before Dox injection could hinder Dox-induced cardiotoxicity; and finally, 4) the combined pretreatment of melatonin with doxorubicin could have a protective effect against hazardous Dox cardiac cell death by hindering ferritinophagy and ferroptosis, by improving antioxidants, and reserving reactive oxygen species (ROS) status [decreasing malondialdehyde (MDA) and increasing reduced glutathione (GSH)].

Ferritinophagy–ferroptosis is one of the recently detected molecular pathways of Dox-induced cardiac muscle cell damage. Despite its wide use as an anticancer drug, its harmful cardiac impact restricts its use, especially in the case of cardiac insufficiency patients. Iron chelators reduce iron overload deposited in various organs, such as the heart. Iron overload facilitates ferritinophagy and ferroptosis by which iron chelators can scavenge cellular accumulation and prevent cardiac death induced by DOX. In addition, ferroptosis depends on the accumulation of ROS and massive lipid accumulation. Therefore, it can be hindered by the use of antioxidants that may modulate lipid peroxidation through glutathione peroxidase 4 (GPx4), which catalyzes the reduction of lipid peroxides in a glutathione-dependent reaction and maintains redox homeostasis (Cui et al., 2015; Mobarra et al., 2016; Fang et al., 2019; Taskin et al., 2019; Yan et al., 2021; Zhai et al., 2021).

Our study assessed Dox-induced acute cardiotoxicity functionally by ECG and ECHO 4 days after Dox injection. ECG showed a decreased heart rate, R and T amplitude, and prolonged QTc with a depressed ST segment. Our results were consistent with many other studies (Durdagi et al., 2021; Ahmed et al., 2021; Abdullah et al., 2019; Jiang et al., 2018; Taskin et al., 2016; Polegato et al., 2015; Dursun et al., 2011; Hazari et al., 2009). On the contrary, Ammar et al. (2011) used a lower Dox dosage (15 mg/kg) injected intraperitoneally as a single dose, revealing cardiotoxicity evidenced by an increased heart rate, elevated ST segment, prolonged QTc interval, and increased T-wave amplitude.

Our ECHO findings showed moderately decreased ejection fraction (EF) and fractional shortening (FS), indicating that acute Dox cardiac toxicity can be detected by ECG changes more accurately than the changes in left ventricle (LV) function detected by measuring the EF and FS. They are more affected by high doses and chronic cumulative Dox dosage that may end up in congestive heart failure and cardiomyopathy (Leonard et al., 2009; Mustafa et al., 2017; Aygun and Gul, 2019; Ma et al., 2019; Sandamali et al., 2019). In line with these explanations, Willis et al. (2019) showed that after the injection of Dox with the same dosage for 7 days, there was a moderate decrease in FS and a moderate increase in the LV systolic diameter with no effect on the diastolic diameter. However, Gioffré et al. (2019) showed ECHO changes in the form of decreased EF and FS with an increase in LVSD and LVDV, but they used a cumulative dose of 24 mg/kg for 2 weeks. Noteworthy, Dox-induced cardiotoxicity is dose-dependent and differs according to the treatment period (Willis et al., 2019).

In similar studies, the Dox-induced acute cardiotoxicity group showed elevated serum levels of cTnI, indicating cardiac muscle cell damage (Mustafa et al., 2017; Ma et al., 2019; Sandamali et al., 2019). In addition, the Dox group showed elevated tissue levels of MDA (Ammar et al., 2011; Dursun et al., 2011; Mustafa et al., 2017; Jiang et al., 2018; Ahmed et al., 2021; Durdagi et al., 2021; Li et al., 2021) and decreased tissue levels of the GSH (Ammar et al., 2011; Dursun et al., 2011; Mustafa et al., 2017; Ahmed et al., 2021). MDA is a stable by-product of lipid peroxidation that can indirectly be used to measure oxidative stress and lipid peroxidation. This confirmed that the oxidative stress and lipid peroxidation load accompanying Dox-induced cardiotoxicity induces ferritinophagy and ferroptosis.

In line with this, we found the upregulation of NCOA4 and IREB2 accompanied by the downregulation of GPX4 (Tadokoro et al., 2020; Li et al., 2021; Wang et al., 2022), SLC7A11 (Ge et al., 2017), and FTH1(ferritin heavy chain one) (Li et al., 2021) in the Dox-induced cardiotoxicity group. NCOA4 and IREB2 are the genes related to the induction of ferritinophagy–ferroptosis and mediate ferritinophagy *via* the activation of autophagic/lysosomal degradation of ferritin, leading to iron cellular accumulation (Masaldan et al., 2018). Moreover, IREB2 regulates the translation of mRNAs that affects iron homeostasis, which is stable when the iron level is normal (Dixon et al., 2012; Ripa et al., 2017). In addition, GPX4 and GSH systems have potent antioxidant activities preventing oxidative stress from playing a key role in lipid peroxidation. Therefore, GPX4 can reduce peroxidized free lipids or even lipids in complex forms, such as phospholipids and membrane lipoproteins, hindering ferroptosis. GSH is essential for maintaining the GPX4 activity, which is also considered a classical ferroptosis inhibitor (Kalinina and Gavriliuk, 2020; Rui et al., 2020). We can conclude that the induction of ferritinophagy accompanied Dox cardiotoxicity in the context of our investigation.

In addition, SLC7A11 is one of the genes involved in iron import and is considered one of the classical ferroptosis inhibitors that is inhibited by excess cytosolic iron. Moreover, it is part of the system XC through which cystine enters the cell to be reduced and utilized for synthesizing GSH (Fang et al., 2020). In addition, FTH1 is one of the genes involved in iron storage, possessing ferroxidase activity to aid iron entry to ferritin (Gao et al., 2016). Hence, according to our outcome, Dox-induced cardiotoxicity induced ferritinophagy and downregulated ferroptosis inhibitors that ended in cellular cardiac death confirmed histopathologically and immunohistochemically.

In the current study, H&E light microscopic examination showed the focal degeneration of the cardiac muscle fibers, blood extravasation, granulation tissue with dense inflammatory infiltration (Öz et al., 2006), and interstitial edema in-between the disrupted cardiomyocytes with increased heart injury scoring (Durdagi et al., 2021). Immunostaining showed the Dox effect on reinforcing apoptosis by the increased expression of caspase-3 (Taskin et al., 2020) and Bax and decreased expression of Bcl2 (Jiang et al., 2018). Likewise, Taskin et al. (2020) concluded that Dox upregulated the protein expression of caspase-3 in a Dox-treated H9c2 cell line. The acute Dox histopathological effect is well-detected within 24 h of its injection as intermuscular edema, myofibrillar loss, infiltration with inflammatory cells, vacuolization, and cardiomyocyte degeneration (Ahmed et al., 2021).

The treated groups in this study were either single-pretreated with melatonin or deferoxamine (Mel-Dox or Des-Dox) or combined with both (Mel-Des-Dox). They displayed improvement that was mainly significant compared with the Dox group, whether it approached the control level or not. However, the best outcome was notably demonstrated by the combo Mel-Des-Dox.

All treated groups led to the downregulation of NCOA4 and IREB2 tissue levels, while the Mel-Des-Dox group restored to the control level. Similarly, all treated groups also led to the upregulation of SLC7A11 and FTH1 tissue levels to the control. At the same time, Mel-Des-Dox showed the best outcome manifested by inhibiting ferritinophagy and improving ferroptosis inhibitors by chelating iron and decreasing oxidative stress, indicating that Dox-induced cardiotoxicity could be hindered.

Regarding GPX4, all treated groups showed significant improvement, in which the Des-Dox group and Mel-Des-Dox showed the best results returning to the control level. Gou et al. (2020) showed the effect of melatonin on the upregulation of GPX4 by improving hypoxic-ischemic brain damage *via* the Akt/Nrf2/Gpx4 signaling pathway. Regarding GSH, the combined treated group Mel-Des-Dox normalized the GSH tissue content to the control level. At the same time, Mel-Dox caused significant improvement without reaching the control level, and the Des-Dox group led to a partially minor improvement. This was similar to Ammar et al. (2011) who showed improvement in GSH levels after using an iron chelator in Dox-induced cardiotoxicity; however, they used deferiprone as an iron chelator for 10 days and injected Dox on day 7. Abd Elkader and Aly (2015) also showed the positive effect of melatonin on iron overload by improving GSH, while they used melatonin subcutaneously in a smaller dose (10 mg/kg/day) for 4 weeks. Guven et al. (2016) showed that the co-administration of Mel and Dox ameliorated oxidative stress and apoptosis generated by Dox on NIH3T3 cells in the culture dish.

The combined treated group Mel-Des-Dox restored the MDA tissue content to the control level. In contrast, Mel-Dox and Des-Dox groups caused significant improvement without reaching the control level. The same result was shown by Ammar et al. (2011) and Abd Elkader and Aly (2015), as discussed previously. In addition, Durdagi et al. (2021) showed that using Mel could improve MDA levels in Dox-induced cardiotoxicity.

Mel-Des-Dox and Des–Dox groups significantly improved cTnI, a protein indicating cardiac myocyte degeneration, reaching the control level (Aygun and Gul, 2019). As a result, treated groups exhibited low iron load levels and lipid peroxidation. In addition, they decreased ferroptosis with improved heart muscle fiber architecture, with combined pretreatment with melatonin and deferoxamine performing the best. As a result, all treatment groups improved heart damage ratings, with the combination group showing the best results. Our results agreed with those of Durdagi et al. (2021) who showed improvement in heart injury using melatonin co-treatment.

Caspase-3, intracellular cysteine-aspartic protease groups (Taskin et al., 2020), is activated upon initiation of apoptosis, and the Bax protein promotes apoptosis. Both showed a significant decrease in all treated groups. In addition, Bcl2, a protein enhancing cell survival by suppressing apoptosis in cells, showed a substantial improvement in all treated groups without returning to the normal level. Li et al. (2020) demonstrated that Dox significantly increased pro-apoptotic Bax and decreased anti-apoptotic Bcl2 protein levels using Western blot analysis, while treatment with Mel for 24 h significantly downregulated Bax and upregulated Bcl2.

Finally, functional assessment by ECG and ECHO showed improved cardiac function in all groups. The best was the combined pretreated groups with an antioxidant (melatonin) and iron chelator (deferoxamine), which indicates that improving oxidative stress, lipid peroxidation, and iron load hinders ferritinophagy and ferroptosis by ameliorating Dox-induced cardiotoxicity. On the contrary, a previous study performed by Durdagi et al. (2021) indicated that melatonin possessed no merit on Dox cardiotoxicity on the ECG changes. However, they used Dox at a higher dose of 45 mg/kg intravenously with melatonin at a lower dose of 10 mg/kg; in addition, it was in combination with Dox not used as a pretreatment. In contrast, we showed improvement in the Mel group that was better augmented by deferoxamine.

In comparison, to the best of our knowledge, our study differed from other protocols. However, there were similarities in specific steps that intersected with our results. Dursun et al. (2011) showed that the oxidative stress of a single dose of Adriamycin cardiotoxicity in rats could be prevented by carnosine, a biological antioxidant. Also, Al-Shabanah et al. (2012) investigated the effect of administration of a single dose of deferoxamine before a single dose of Dox resulted in a complete reversal of Dox-induced alteration in cardiac enzymes and gene expression of TGF-*β*, Smad2, Smad4, CDKN2A, p53, and Smad7 and Mdm2 mRNA expression levels to normal levels. Fang et al. (2019) also showed that Dox-induced cardiomyopathy was due to the ferroptosis pathway that can be prevented by pretreatment with an iron chelator, dexrazoxane, and ferroptosis inhibitor, ferrostatin-1, through the assessment of the Nrf2/Hmox1 pathway. Kumfu et al. (2018) showed that combining an iron chelator (deferiprone) with an antioxidant (N-acetylcysteine) exerts greater efficacy on cardio-protection. Also, Hijazi et al. (2019) showed that antioxidants and anti-inflammatory agents (*Achillea fragrantissima*) improve cardiac enzymes and inflammatory and oxidative stress in Dox-induced cardiotoxicity. Li et al. (2021) investigated the cardioprotective role of the antioxidant fisetin and its mechanism of action in Dox-induced cardiomyopathy rats and H9c2 cell models. They showed that fisetin treatment could markedly ameliorate Dox-induced cardiotoxicity and improve cardiac function by attenuating ferroptosis of cardiomyocytes by reducing MDA and increasing GSH levels (Ma et al., 2019), leading to reversing the decrease in the GPX4 level and upregulation of FTH1 genes.

## 5 Conclusion

Pretreatment with an antioxidant, melatonin (Mel), and an iron chelator, deferoxamine (Des), could alleviate acute Dox-induced cardiac cell damage by preventing ferritinophagy and ferroptosis, preserving cardiac functions. In addition, deferoxamine (Des) has a short plasma half-life of about 20–30 min, as reviewed by Cui et al. (2015)and Farr and Xion (2020). Therefore, it may need to be repeatedly injected using multiple doses before regular Dox injection as anticancer treatment. However, long-term side effects of deferoxamine include visual and auditory neurotoxicity due to chronic treatment, and acute effects include abdominal pain, diarrhea, nausea, vomiting, and hypotension, as stated by Arandi et al. (2015)and Cui et al. (2015), that are dose-dependent. Therefore, using melatonin could help reduce these side effects as it improves the Des effect without using multiple or high dosages, helping in avoiding side effects. So, further studies are warranted to adjust the best dose.

## Data Availability

The original contributions presented in the study are included in the article/Supplementary Materials, further inquiries can be directed to the corresponding author.
