# Supplementary figures and images for: Synergistic cardioprotective effects of melatonin and deferoxamine through the improvement of ferritinophagy in doxorubicin-induced acute cardiotoxicity
Source: Front Physiol. 2022 Nov 30;13:1050598. doi: 10.3389/fphys.2022.1050598 (PMC9748574; doi:10.3389/fphys.2022.1050598)

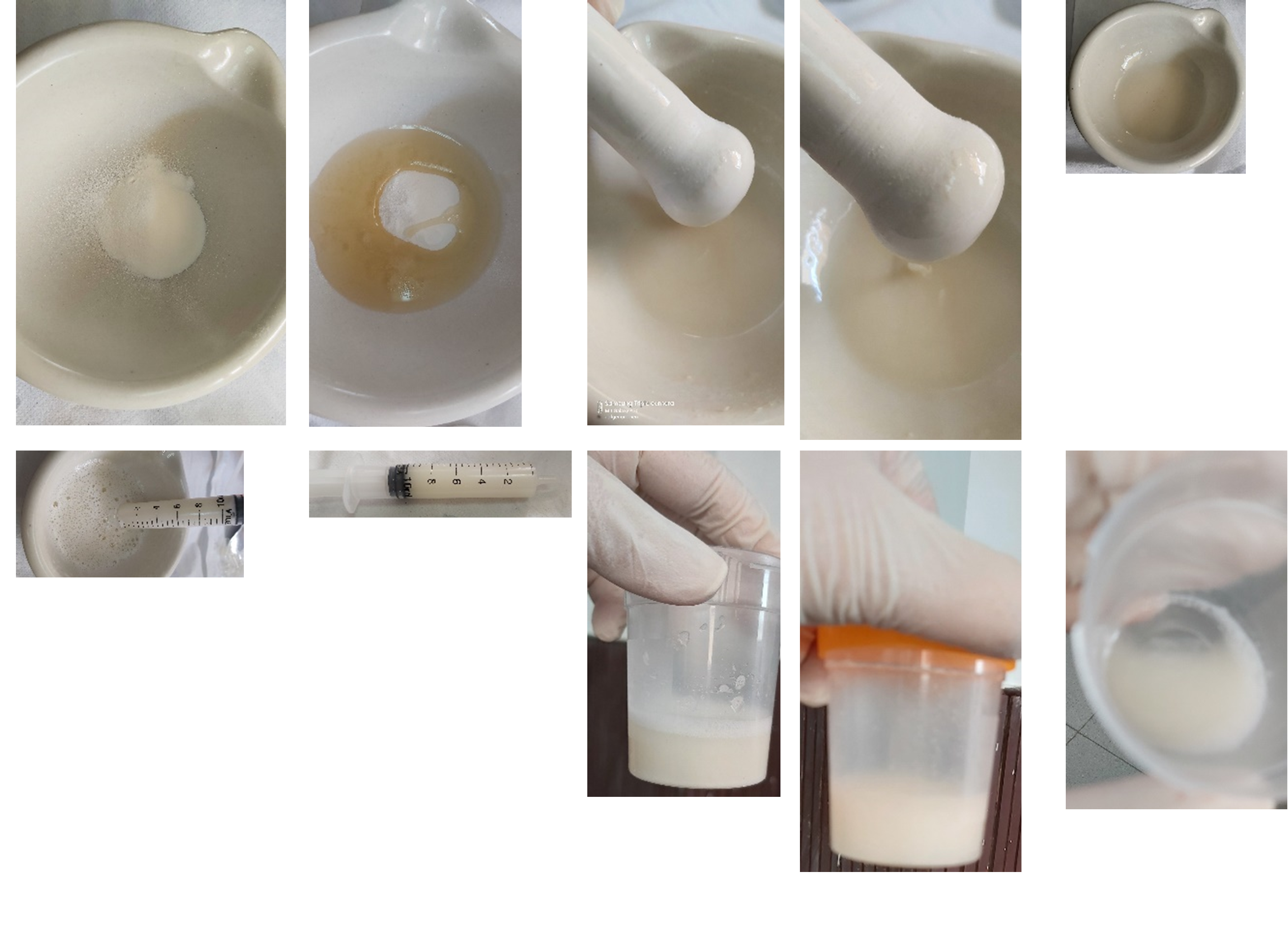

Supplement: Supplementary file 1 [file Image6.TIF]

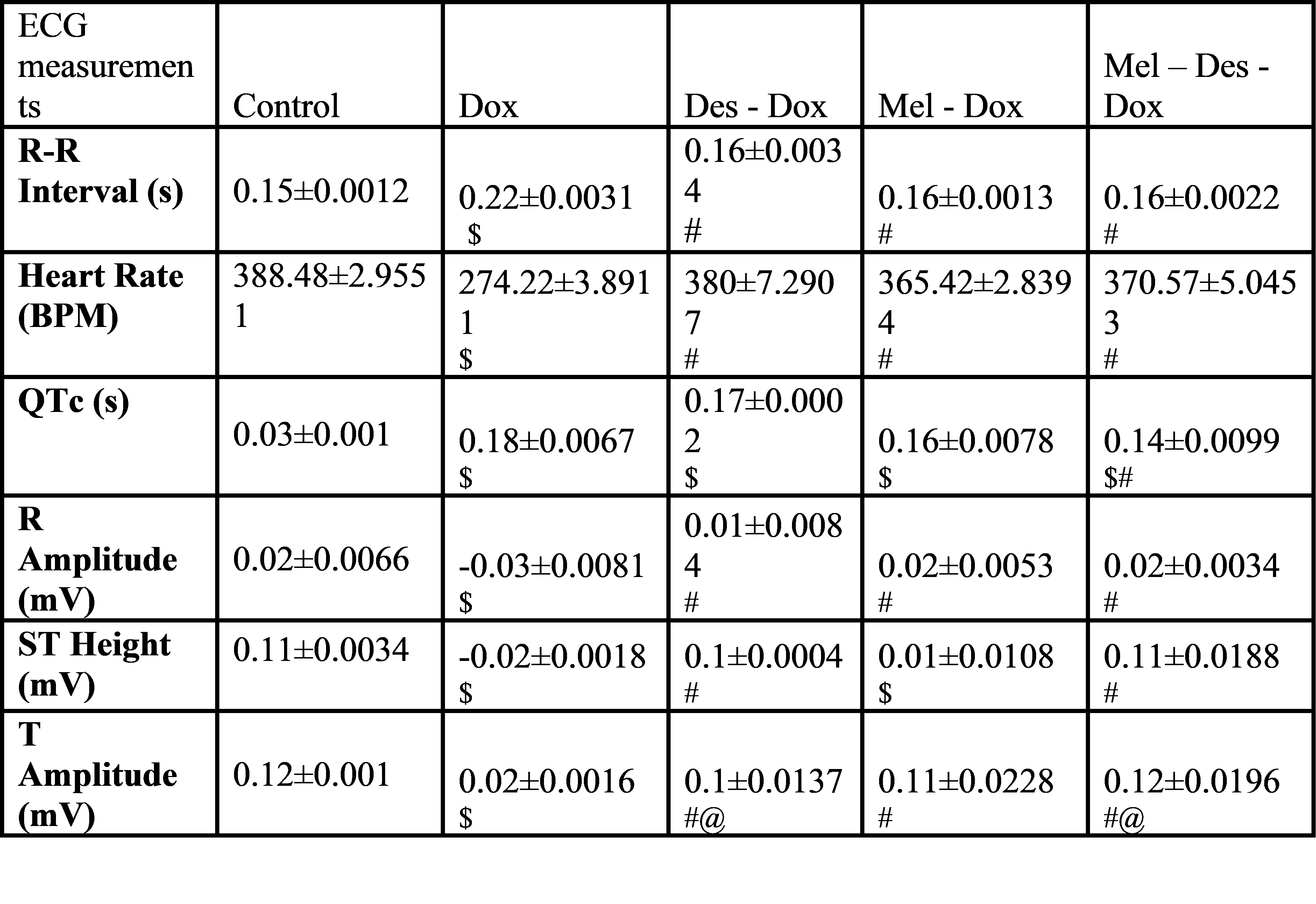

Supplement: Supplementary file 3 [file Image3.TIF]

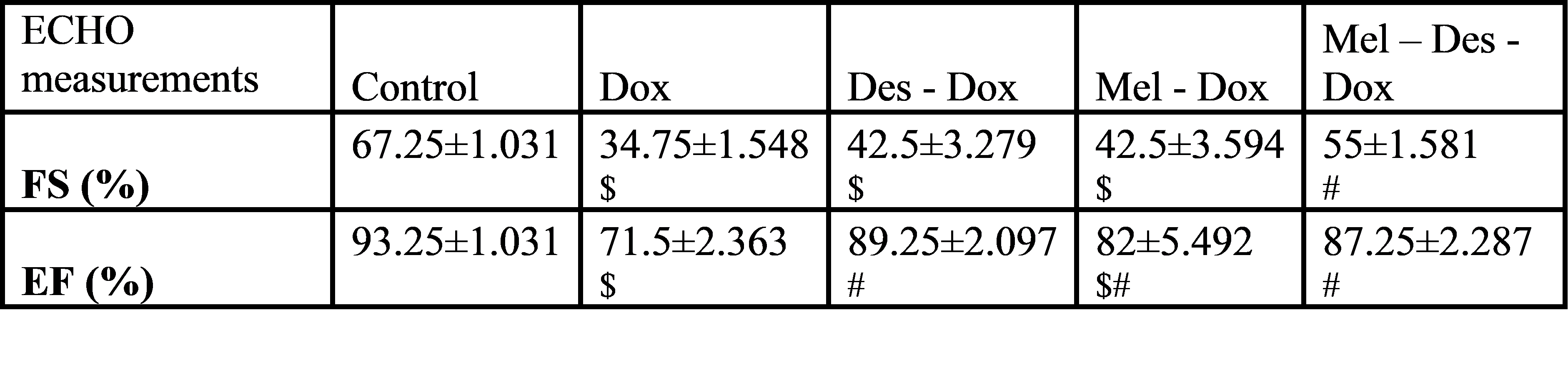

Supplement: Supplementary file 4 [file Image4.TIF]

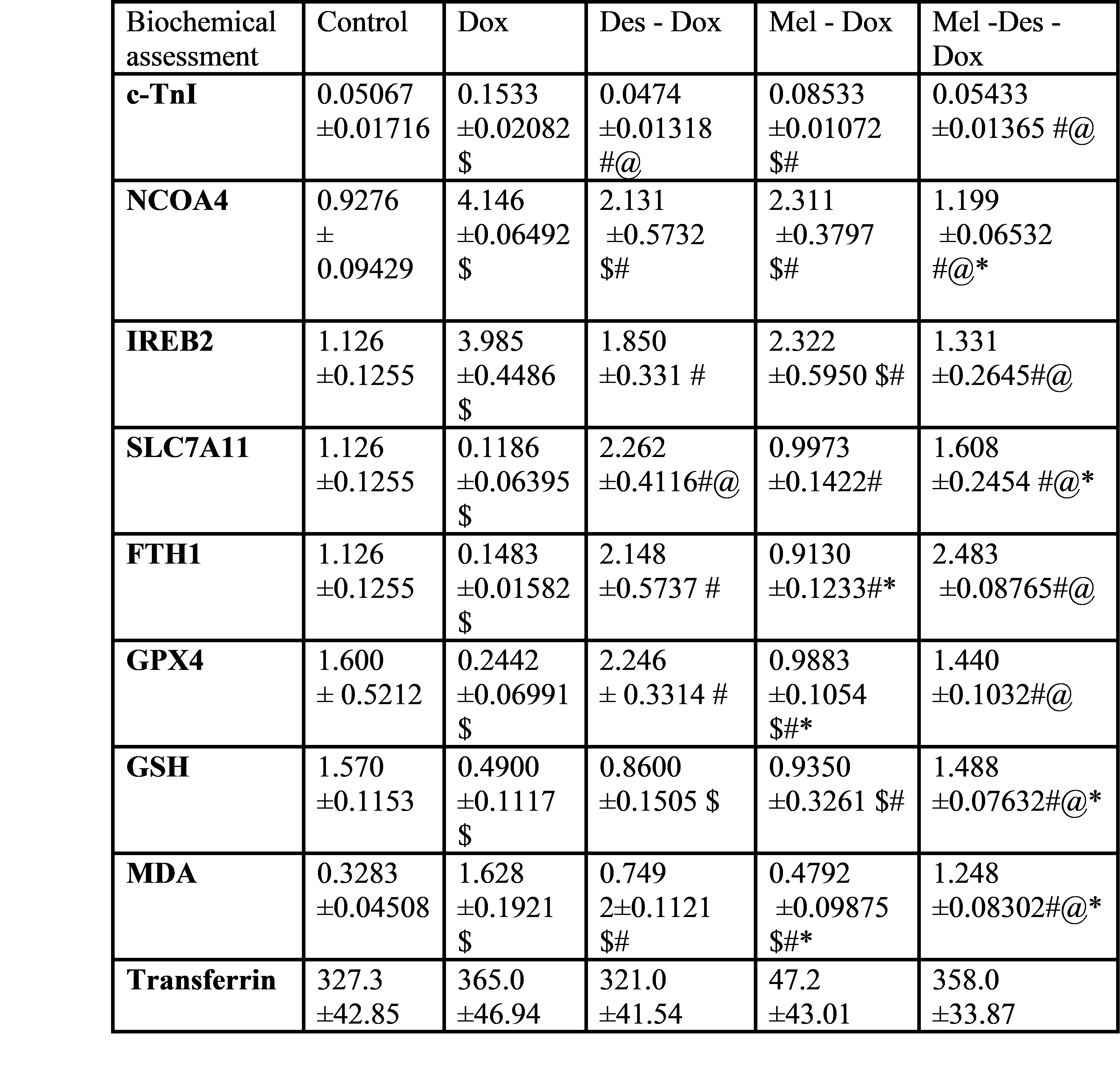

Supplement: Supplementary file 5 [file Image1.TIF]

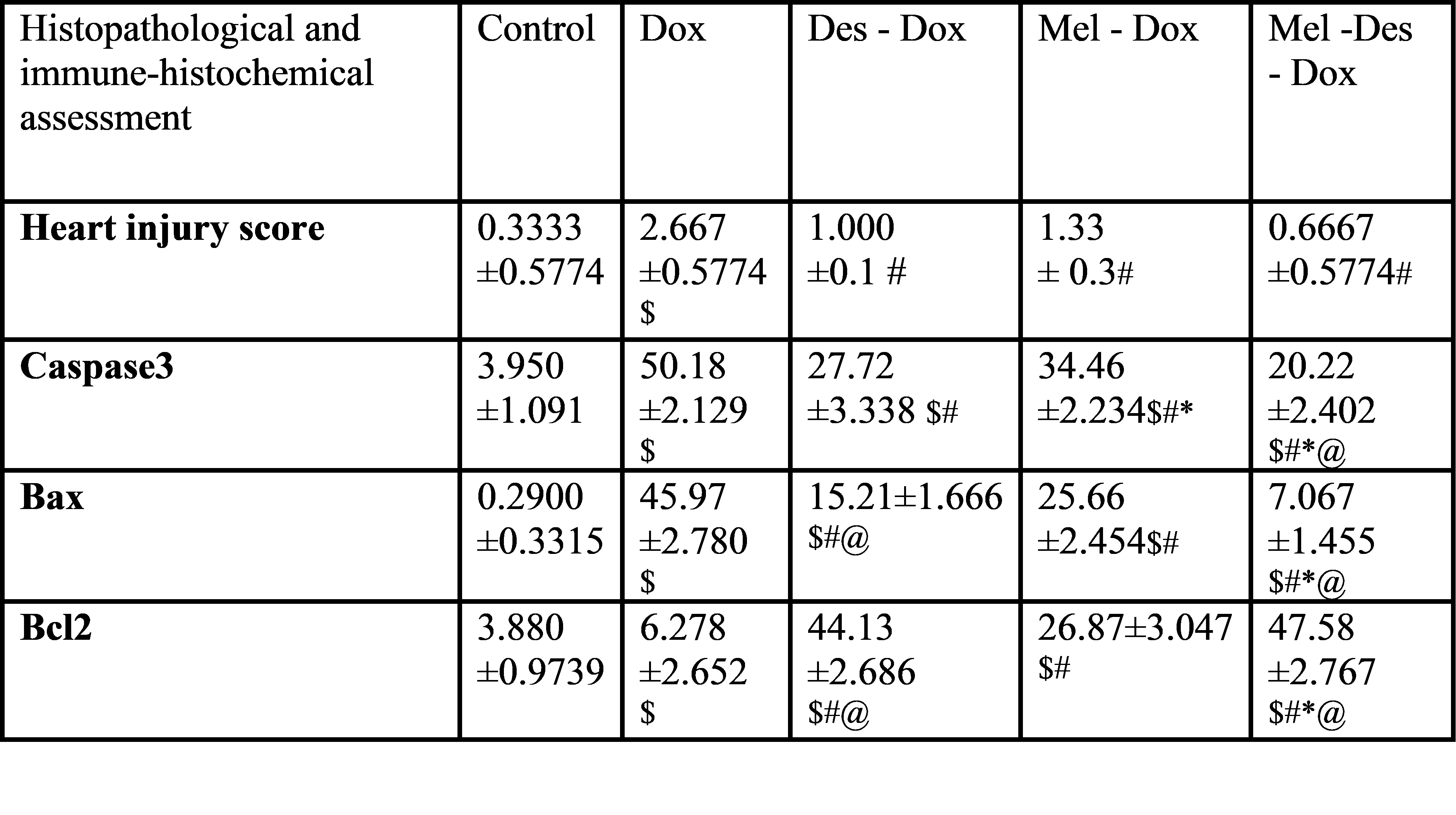

Supplement: Supplementary file 7 [file Image2.PNG]

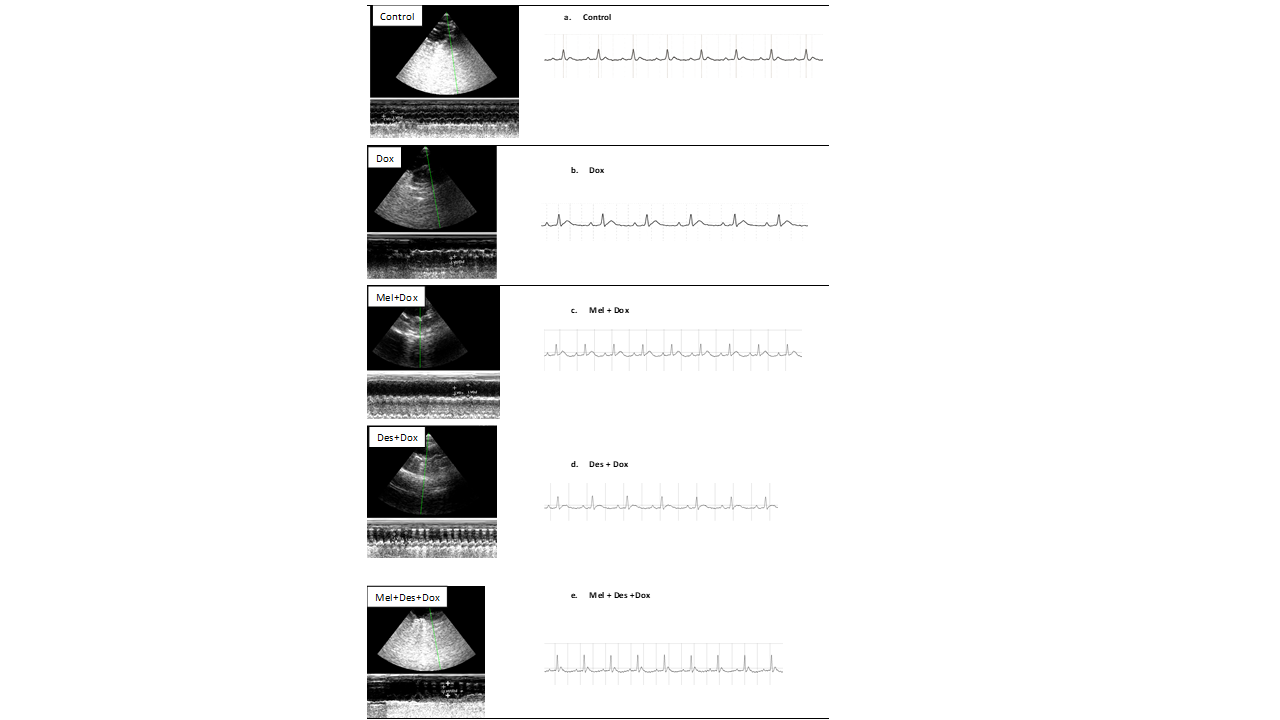

Supplement: Supplementary file 8 [file Image5.TIF]
