# Supplementary material for: Synergistic cardioprotective effects of melatonin and deferoxamine through the improvement of ferritinophagy in doxorubicin-induced acute cardiotoxicity
Source: Front Physiol. 2022 Nov 30;13:1050598. doi: 10.3389/fphys.2022.1050598 (PMC9748574; doi:10.3389/fphys.2022.1050598)
Supplement: Supplementary file 2 [file Table1.DOCX]

**Figures Legends**

**Figure 1:** Timeline of drug administration throughout the experiment. Dox: doxorubicin, Mel: melatonin, Des: Deferoxamine.

**Figure 2 (a – h):** shows Biochemical assay results for cTnI, NCOA4, IREB2, SLC7AII, FTH1, GPX4, GSH, and MDA respectively. $: significant versus control, #: significant versus Dox, @: significant versus Mel-Dox, *: significant versus Des-Dox. Mel: melatonin, Des: Deferoxamine, Dox: Doxorubicin Mel: melatonin, Des: Deferoxamine, Dox: Doxorubicin.

**Figure 3:** (a) Representative figures of H&E-stained sections of the different study groups displaying normal cardiac myocytes in the control, (Mel) and (Des) groups. The (Dox) group shows focal degeneration of the cardiac muscle fibers with cardiomyocyte fragmentation (f) and blood extravasation (Ex). Granulation tissue infiltrated with dense inflammatory infiltration (I) formed of lymphocytes, neutrophils and macrophages is also observed. Dilated congested capillaries (C) and interstitial edema (Ed) in-between the disrupted cardiomyocytes are also noticed. The (Mel-Dox) group shows minimal myocyte disruption with residual mononuclear inflammatory cellular infiltration (I). The (Des-Dox) group shows regularly-arranged cardiac muscle fiber with minimal blood extravasation (Ex). The combined (Mel-Des-Dox) group shows improved architecture of the cardiac muscle fibers where they exhibit normal acidophilic sarcoplasm, central oval vesicular cardiomyocyte nuclei and peripheral flat spindle-shaped nuclei of fibroblasts. The cardiomyocytes appear separated by narrow inter-fibrillar spaces and healthy blood capillaries. (Scale bar 50µm) (b) Heart injury score of the different study groups. ($: significant versus control, #: significant versus Dox, @: significant versus Mel-Dox, *: significant versus Des-Dox at p<0.05, (n =10) using ANOVA, Tukey post-hoc test for pairwise comparison.). Mel: melatonin, Des: Deferoxamine, Dox: Doxorubicin.

**Figure 4:** Representative figures of caspase-3 immunohistochemical expression in the myocardium. The (a) Control group, (b) Mel, (c) Des, show negative caspase-3 immunoreactivity in the myocardial fibers. (d) Dox group shows strong positive caspase-3 immune reaction in the cardiac myofibrils. (e) Mel-Dox and (f) Des-Dox show moderate caspase-3 immune reaction in the cardiomyocytes. (g) Minimal immunoreactivity is noticed in the combined Mel and Des-treated group (Mel-Dox-Des) (scale bar 50µm). (h) Area percentage of caspas3 immunohistochemical expression in the different study groups, $: significant versus control, #: significant versus Dox, @: significant versus Mel-Dox, *: significant versus Dox-Des at p<0.05, (n =10) using ANOVA, Tukey post-hoc test for pairwise comparison. Mel: melatonin, Des: Deferoxamine, Dox: Doxorubicin.

**Figure 5**: (a) Representative figures of Bax/Bcl2 immunoreactivity in the myocardium (scale bar 50µm). (h) Area percentage of Bax/Bcl2 immunohistochemical expression in the different study groups, $: significant versus control, #: significant versus Dox, @: significant versus Mel-Dox, *: significant versus Des-Dox at p<0.05, (n =10) using ANOVA, Tukey post-hoc test for pairwise comparison. Mel: melatonin, Des: Deferoxamine, Dox: Doxorubicin.

**Figure 6:** The graphs are showing the statistical difference between groups regarding ECG measurements. $: significant versus control, #: significant versus Dox, @: significant versus Mel-Dox, *: significant versus Des-Dox. Mel: melatonin, Des: Deferoxamine, Dox: Doxorubicin Mel: melatonin, Des: Deferoxamine, Dox: Doxorubicin.

**Figure 7:** The graphs are showing the results of Ejection fraction and fractional shortening. $: significant versus control, #: significant versus Dox, @: significant versus Mel-Dox, *: significant versus Des-Dox. Mel: melatonin, Des: Deferoxamine, Dox: Doxorubicin Mel: melatonin, Des: Deferoxamine, Dox: Doxorubicin.

**Supplementary Figure 8:** Echocardiography and ECG records of (a) control (b) Dox (c) Mel-Dox (d) Des-Dox (e) Mel-Des-Dox. Mel: melatonin, Des: Deferoxamine, Dox: Doxorubicin Mel: melatonin, Des: Deferoxamine, Dox: Doxorubicin.

**Supplementary Figure 9:** Steps of Melatonin preparation in our lab.
