# Supplementary material for: Synergistic cardioprotective effects of melatonin and deferoxamine through the improvement of ferritinophagy in doxorubicin-induced acute cardiotoxicity
Source: Front Physiol. 2022 Nov 30;13:1050598. doi: 10.3389/fphys.2022.1050598 (PMC9748574; doi:10.3389/fphys.2022.1050598)
Supplement: Supplementary file 6 [file Table2.DOCX]

**Supplementary Tables:**

**Table 1: shows biochemical assessment statistical results**

| Biochemical assessment | Control | Dox | Des - Dox | Mel - Dox | Mel -Des - Dox |
| --- | --- | --- | --- | --- | --- |
| **c-TnI** | 0.05067  ±0.01716 | 0.1533  ±0.02082 $ | 0.0474  ±0.01318 #@ | 0.08533  ±0.01072 $# | 0.05433  ±0.01365 #@ |
| **NCOA4** | 0.9276  ± 0.09429 | 4.146  ±0.06492 $ | 2.131  ±0.5732 $# | 2.311  ±0.3797  $# | 1.199  ±0.06532  #@* |
| **IREB2** | 1.126  ±0.1255 | 3.985  ±0.4486 $ | 1.850  ±0.331 # | 2.322  ±0.5950 $# | 1.331  ±0.2645#@ |
| **SLC7A11** | 1.126  ±0.1255 | 0.1186  ±0.06395 $ | 2.262  ±0.4116#@ | 0.9973  ±0.1422# | 1.608  ±0.2454 #@* |
| **FTH1** | 1.126  ±0.1255 | 0.1483  ±0.01582 $ | 2.148  ±0.5737 # | 0.9130 ±0.1233#* | 2.483  ±0.08765#@ |
| **GPX4** | 1.600  ± 0.5212 | 0.2442  ±0.06991 $ | 2.246  ± 0.3314 # | 0.9883  ±0.1054 $#* | 1.440  ±0.1032#@ |
| **GSH** | 1.570  ±0.1153 | 0.4900  ±0.1117 $ | 0.8600  ±0.1505 $ | 0.9350  ±0.3261 $# | 1.488  ±0.07632#@* |
| **MDA** | 0.3283  ±0.04508 | 1.628  ±0.1921 $ | 0.749  2±0.1121 $# | 0.4792  ±0.09875 $#* | 1.248  ±0.08302#@* |
| **Transferrin** | 327.3  ±42.85 | 365.0  ±46.94 | 321.0  ±41.54 | 47.2  ±43.01 | 358.0  ±33.87 |

Mean ±SD for cTnI, NCOA4, IREB2, SLC7AII, FTH1, GPX4, GSH, and MDA respectively. $: significant versus control, #: significant versus Dox, @: significant versus Mel-Dox, *: significant versus Des-Dox. Mel: melatonin, Des: Deferoxamine, Dox: Doxorubicin Mel: melatonin, Des: Deferoxamine, Dox: Doxorubicin.

**Table 2: shows histopathological and immune-histochemical assessment statistical results**

| Histopathological and immune-histochemical assessment | Control | Dox | Des - Dox | Mel - Dox | Mel -Des - Dox |
| --- | --- | --- | --- | --- | --- |
| **Heart injury score** | 0.3333  ±0.5774 | 2.667  ±0.5774 $ | 1.000  ±0.1 # | 1.33  ± 0.3# | 0.6667  ±0.5774# |
| **Caspase3** | 3.950  ±1.091 | 50.18  ±2.129 $ | 27.72  ±3.338 $# | 34.46  ±2.234$#* | 20.22  ±2.402  $#*@ |
| **Bax** | 0.2900  ±0.3315 | 45.97  ±2.780 $ | 15.21±1.666  $#@ | 25.66  ±2.454$# | 7.067  ±1.455 $#*@ |
| **Bcl2** | 3.880  ±0.9739 | 6.278  ±2.652  $ | 44.13  ±2.686  $#@ | 26.87±3.047  $# | 47.58 ±2.767  $#*@ |

Mean ±SD for Heart injury scor, Caspase 3, Bax and BCL2. $: significant versus control, #: significant versus Dox, @: significant versus Mel-Dox, *: significant versus Des-Dox. Mel: melatonin, Des: Deferoxamine, Dox: Doxorubicin Mel: melatonin, Des: Deferoxamine, Dox: Doxorubicin.

**Table 3: shows ECG measurements statistical results**

| ECG measurements | Control | Dox | Des - Dox | Mel - Dox | Mel – Des -Dox |
| --- | --- | --- | --- | --- | --- |
| **R-R Interval (s)** | 0.15±0.0012 | 0.22±0.0031  $ | 0.16±0.0034  # | 0.16±0.0013  # | 0.16±0.0022  # |
| **Heart Rate (BPM)** | 388.48±2.9551 | 274.22±3.8911  $ | 380±7.2907  # | 365.42±2.8394  # | 370.57±5.0453  # |
| **QTc (s)** | 0.03±0.001 | 0.18±0.0067  $ | 0.17±0.0002  $ | 0.16±0.0078  $ | 0.14±0.0099  $# |
| **R Amplitude (mV)** | 0.02±0.0066 | -0.03±0.0081  $ | 0.01±0.0084  # | 0.02±0.0053  # | 0.02±0.0034  # |
| **ST Height (mV)** | 0.11±0.0034 | -0.02±0.0018  $ | 0.1±0.0004  # | 0.01±0.0108  $ | 0.11±0.0188  # |
| **T Amplitude (mV)** | 0.12±0.001 | 0.02±0.0016  $ | 0.1±0.0137  #@ | 0.11±0.0228  # | 0.12±0.0196  #@ |

Mean ±SD for ECG measurements. $: significant versus control, #: significant versus Dox, @: significant versus Mel-Dox, *: significant versus Des-Dox. Mel: melatonin, Des: Deferoxamine, Dox: Doxorubicin Mel: melatonin, Des: Deferoxamine, Dox: Doxorubicin.

**Table 4: shows ECHO measurements statistical results**

| ECHO measurements | Control | Dox | Des - Dox | Mel - Dox | Mel – Des - Dox |
| --- | --- | --- | --- | --- | --- |
| **FS (%)** | 67.25±1.031 | 34.75±1.548  $ | 42.5±3.279  $ | 42.5±3.594  $ | 55±1.581  # |
| **EF** **(%)** | 93.25±1.031 | 71.5±2.363  $ | 89.25±2.097  # | 82±5.492  $# | 87.25±2.287  # |

Mean ±SD for ECHO measurements. $: significant versus control, #: significant versus Dox, @: significant versus Mel-Dox, *: significant versus Des-Dox. Mel: melatonin, Des: Deferoxamine, Dox: Doxorubicin Mel: melatonin, Des: Deferoxamine, Dox: Doxorubicin.
